# The effect of LDL-C status on the association between increased coronary artery calcium score and compositional plaque volume progression in statins-treated diabetic patients: evaluated using serial coronary CTAs

**DOI:** 10.1186/s12933-022-01556-y

**Published:** 2022-06-30

**Authors:** Rui Shi, Yue Gao, Li-Ling Shen, Ke Shi, Jin Wang, Li Jiang, Yuan Li, Zhi-Gang Yang

**Affiliations:** Department of Radiology, West China Hospital, Sichuan University, 37# Guo Xue Xiang, Chengdu, 610041 Sichuan China

**Keywords:** Diabetes mellitus, Coronary computed tomography angiography, Coronary atherosclerosis, Statins, LDL-C

## Abstract

**Background:**

In statins-treated diabetic mellitus (DM) patients, longitudinal coronary CTA (CCTA) evidence is scarce regarding the relationship between coronary Agatston artery calcification scores (CACs) and coronary plaque progression. This study was designed to investigate whether the association between CACs progression and compositional plaque volumes (PVs) progression differed between follow-up low-density lipoprotein cholesterol (LDL-C) controlled and uncontrolled groups in statins-treated DM patients.

**Methods:**

From January 2015 to June 2021, 208 patients who submitted serial clinically indicated CCTAs in our hospital were included in this study. Participants were further subdivided into LDL-C controlled (n = 75) and LDL-C uncontrolled (n = 133) groups according to whether the LDL-C reached the treatment goals at follow-up. Baseline and follow-up CCTA image datasets were quantified analysis at per-patient and per-plaque levels. The annual change of total PV (TPV), calcific PV(CPV), non-calcific PV (NCPV), low-density non-calcific PV (LD-NCPV), and CACs were assessed and further compared according to follow-up LDL-C status. The effect of CACs progression on the annual change of componential PVs was evaluated according to follow-up LDL-C status at both per-patient and per-plaque levels.

**Results:**

The annual change of CACs was positively associated with the annual change of TPV (β = 0.43 and 0.61, both p < 0.001), CPV (β = 0.23 and β = 0.19, p < 0.001 and p = 0.004, respectively), NCPV (β = 0.20 and β = 0.42, p < 0.001 and p = 0.006, respectively), and LD-NCPV (β = 0.08 and 0.13, p < 0.001 and p = 0.001, respectively) both on per-patients and per-plaque levels. LDL-C status had no effect on the annual change of TPV, CPV, NCPV, and LD-NCPV (all p > 0.05). After adjusting for confounding factors, on the per-patient level, the increase in CACs was independently associated with annual change of TPV (β = 0.650 and 0.378, respectively, both p < 0.001), CPV (β = 0.169 and 0.232, respectively, p = 0.007 and p < 0.001), NCPV (β = 0.469 and 0.144, respectively, both p = 0.001), and LD-NCPV (β = 0.082 and 0.086, respectively, p = 0.004 and p = 0.006) in LDL-C controlled and LDL-C uncontrolled group. On the per-plaque level, the increase in CACs was independently associated with the annual change of NCPV and LD-NCPV in LDL-C uncontrolled patient (β = 0.188 and 0.106, p < 0.001), but not in LDL-C controlled group (β = 0.268 and 0.056, p = 0.085 and 0.08).

**Conclusions:**

The increase of CACs in statins-treated DM patients indicates the progression of compositional PVs. From a per-plaque perspective, there might be increased instability of individual plaques concomitant with CACs increase in LDL-C uncontrolled patients.

**Supplementary Information:**

The online version contains supplementary material available at 10.1186/s12933-022-01556-y.

## Background

Coronary artery disease (CAD) is the leading cause of morbidity and life years lost worldwide [1]. Low-density lipoprotein cholesterol (LDL-C) deposition in arterial walls is an important part of coronary atherosclerosis formation [2]. Comorbidities of diabetes mellitus (DM), including insulin resistance, hyperglycemia and hyperlipidemia, have synergistically contributed to the development and advancement of CAD [3]. Considerable evidence suggests that serum LDL-C is a predictor of CAD in the DM population, even exceeding the predictive power of hemoglobin A1c (HbA1c) [4]. Statins are the cornerstone of lipid-lowering drugs in DM. Once diabetes is diagnosed, patients with no contraindications are routinely recommended lipid therapy as the primary prevention of cardiovascular disease [5]. Due to poor patient compliance, not all DM patients maintain ideal lipid control at follow-up.

Several studies have focused on the natural history of coronary atherosclerosis progression [6–8]. Coronary artery calcium score (CACs) is considered one of the critical indicators for coronary atherosclerosis severity, but the results maintain discrepancy in individuals on medication, especially with statins use. Coronary CTA (CCTA) is an important means to assess coronary atherosclerosis with the ability to assess both plaque composition and stenosis [9]. Longitudinal CCTA evidence on the association between CACs and coronary plaque progression in DM patients is scarce. The present study was designed to investigate the impactors of compositional plaque volumes (PVs) progression in statins-treated DM patients and to explore whether the association between CACs progression and compositional PVs progression differed between increased follow-up LDL-C and normal LDL-C groups in statins-treated DM patients who underwent serial CCTAs.

## Methods

### Study population

The Institutional Review Board approved this study, and informed consent was waived due to the retrospective nature of this investigation. From January 2015 to June 2021, clinically diagnosed diabetes patients who received clinically indicated serial CCTAs in our hospital were retrospectively recruited. Baseline CCTAs were performed for angina, suspected angina, abnormal ECGs, preoperative evaluation, or screening of CAD in the population with multiple CAD high-risk factors. Follow-up CCTA examinations were performed for new developed cardiovascular symptoms, worsening of pre-existing cardiovascular symptoms, or regular physical exams. Inclusion criteria were (1) at least a one-year interscan interval between serial CCTAs; (2) laboratory tests within one week before and after each CCTA scan; (3) indications for statin use and withdrawal from statins for no more than three months during the follow-up period. Exclusion criteria were (1) revascularization or coronary artery bypass during two CCTA scans, (2) severe coronary calcification (CAC score > 1000), (3) documented cardiac surgery (e.g., valve replacement, any surgery of the heart or arteries), (4) poor image quality (image quality score less than 2), and (5) lack of crucial information (e.g., basic clinical information, statins use, baseline or follow-up lipid profile).

**Fig. 1:**
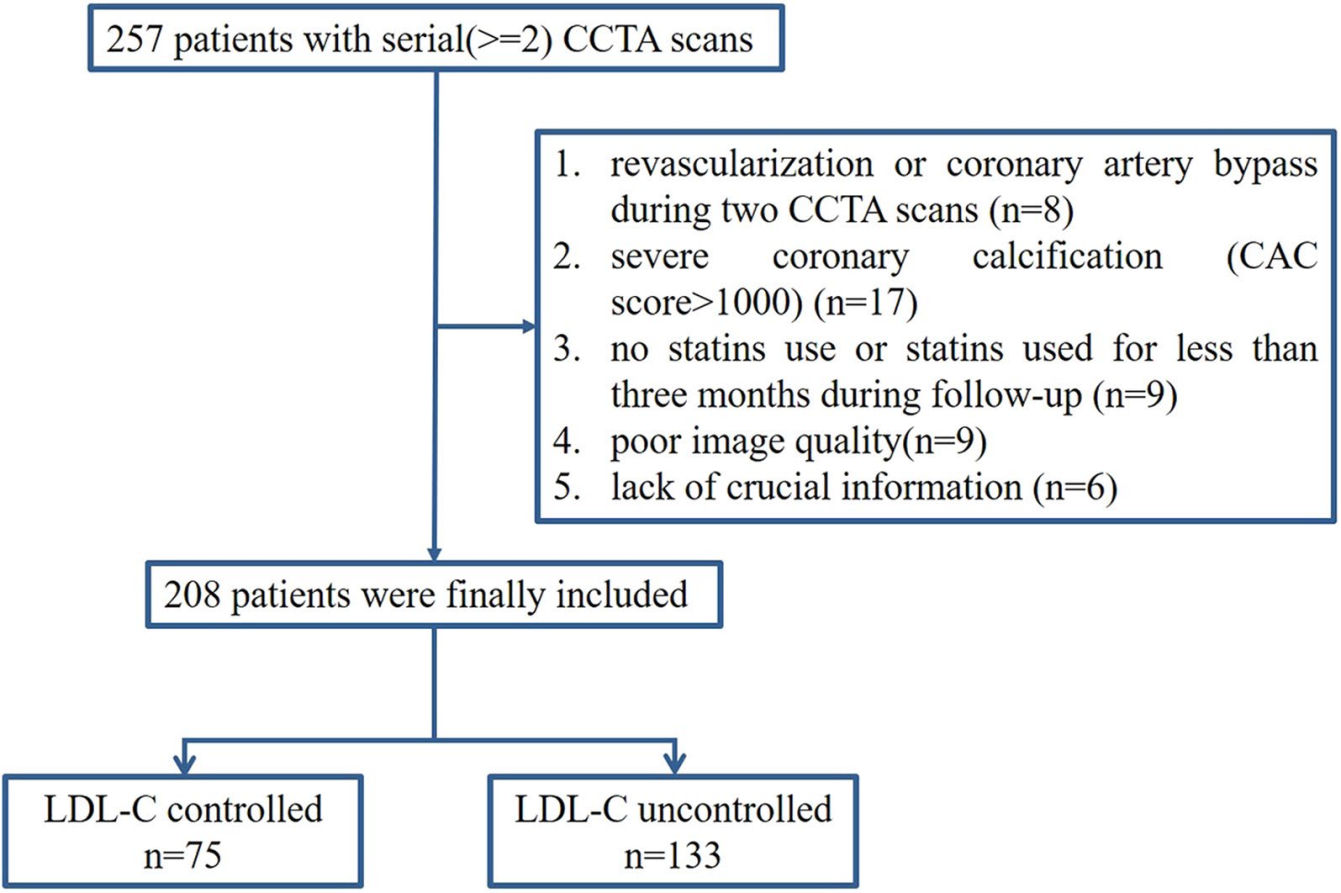
Flow chart of the study. LDL-C: low-density lipoprotein cholesterol; CCTA: Coronary computed tomographic angiography

After using the inclusion and exclusion criteria, 208 patients were finally included in the present research. Atherosclerotic cardiovascular disease (ASCVD) risk categories and corresponding LDL-C treatment goals were assessed according to the AACE/ACE consensus statement [10]. Participants were further subdivided into LDL-C controlled (n = 75) and LDL-C uncontrolled (n = 133) groups according to whether the LDL-C achieved individual’s treatment goals at follow-up (Fig. 1, Additional file 1: Table S1).

Clinical histories, medication records, and laboratory examinations results were obtained through Hospital Information System and Laboratory Information System before the baseline and follow-up coronary CTA. Statins use during follow-up was assessed in follow-up medical records.

### Coronary CTA obtain and analysis

#### Coronary CTA scan protocol

CCTAs data acquisition and image post-processing were performed in accordance with the Society of Cardiovascular Computed Tomography guidelines [11]. All CCTA scans were performed on multidetector CT systems, and beta-blocker was not routinely used in our study. The scanning range was 20 mm below the inferior cardiac apex from the tracheal bifurcation. Detailed scan parameters are displayed in supplementary materials (Additional file 1: Table S2).

#### Coronary CTA analysis

**Fig. 2:**
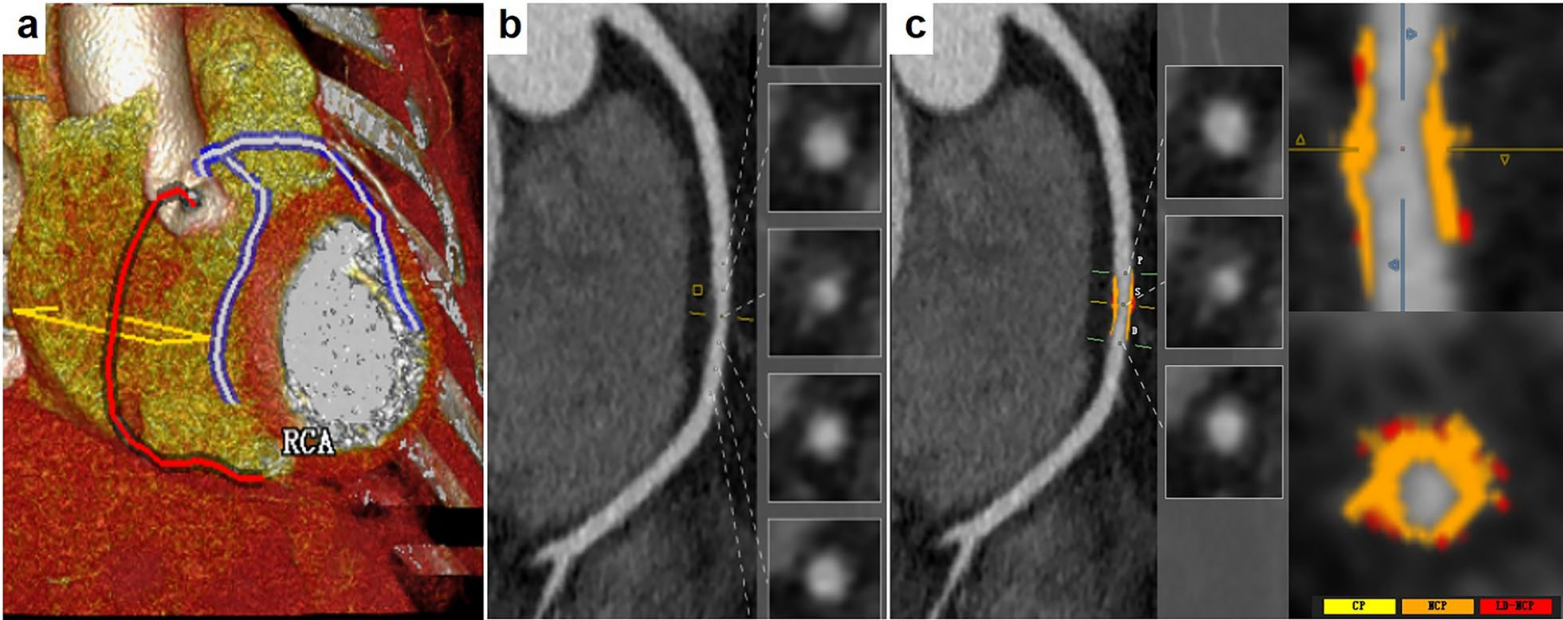
Schematic diagram of quantitative coronary plaque analysis. After a landmark in the ascending aorta is placed, coronary centerlines generation automatically (**a** red and light purple lines). A surface reconstruction image of the target vessel is automatically generated (**b**), and the stenosis marker and lesion range are defined on the surface reconstruction images. After the plaque quantitative analysis is completed, different plaque components are marked with colors (**c**, yellow for calcified plaque, orange for non-calcified plaque, and red for low-density non-calcified plaque)

**Fig. 3:**
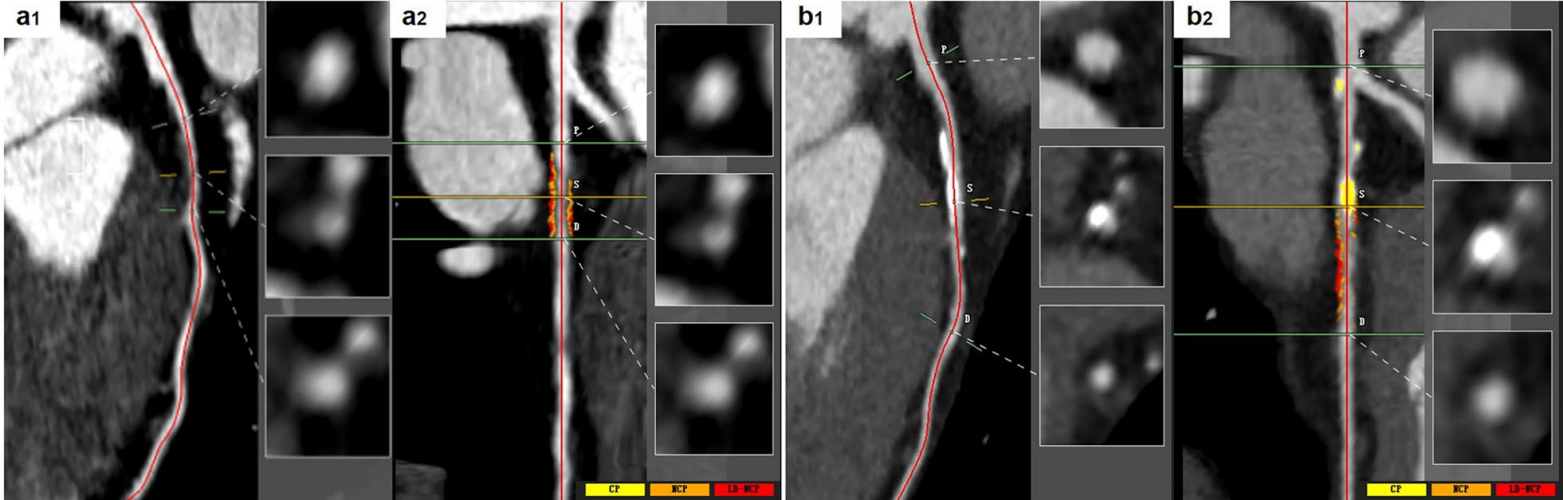
Representative imaging. A 48-year-old female diabetic patient whose ASCVD risk category is very high risk. There was a non-calcific plaque on the proximal left anterior descending coronary artery at the baseline CCTA (**a1**, **a2**). The non-calcified plaque volume was 107.21 mm^3^, and the stenosis degree was 30.26%. After an 18-month follow-up, the LDL-C at follow-up was 3.6 mmol/L. Follow-up CCTA showed an increase in the extent of the original lesion, along with new formation of calcified components. The calcified volume in the plaque was 97.4 mm^3^ and the non-calcified volume was 144.24 mm^3^, with a stenosis of 65.5% (**b1**, **b2**)   .

Baseline and follow-up datasets were analyzed by two experienced radiologists (with at least 3–4 years of experience performing CCTA analysis) who were blinded to all clinical data. Image quality was independently evaluated by the two radiologists using a 4-point scale system [12]. The 4-point scale was as follows: 4, excellent, no artifacts; 3, good, mild artifacts and do not affect the analysis of coronary atherosclerotic lesions; 2, acceptable, moderate artifact present but images are still interpretable; and 1, poor, completely uninterpretable image quality due to severe artifacts. Image datasets were post-processed and quantitatively analyzed by the two radiologists using commercial software (cvi42, Circle Cardiovascular Imaging, Calgary, Canada). Coronary were evaluated using a modified 17-segment American Heart Association model, and segments with a diameter ≥ 2 mm were included analysis. Atherosclerotic plaques were matched between baseline and follow-up coronary trees using branch points, distance from ostia, or branch vessel takeoffs as landmarks. CCTA image datasets were loaded in the coronary module, a landmark was placed in the ascending aorta, and coronary centerlines is generated automatically, with manual corrections as needed. Coronary vessels visually and quantitatively assessment was performed based on existing centerlines. After defining the stenosis marker and lesion range, stenosis measurements, total PV (TPV), calcific PV(CPV), non-calcific PV (NCPV), and low-density non-calcific PV (LD-NCPV) were obtained (Fig. 2). Non-contrast CCTA imaging was used for the Calcium Scoring post-processing with a threshold of 130 HU and a pre-set calcification mass calibration factor. After defining the range of calcifications, CACs were automatically calculated using the Agatston method. Annual change of PVs was defined as total PV change (mm^3^) divided by the inter-scan period (year). Annual change of CACs was defined as total CACs change divided by the inter-scan period (year). The analysis was performed on a per-plaque basis, and the whole-heart data was the sum of individual plaques in the coronary tree. When the distance between two lesions was less than 5 mm, it was regarded as one plaque, and when the distance was greater than 5 mm, it was regarded as two separate serial lesions. Obstructive lesions were defined as area stenosis greater than 50%. Patients with obstructive lesions in the coronary artery tree were defined as obstructive patients. A representative imaging is shown in Fig. 3.

#### Reproducibility analysis

To determine intra- and inter-observer variability, stenosis, compositional PVs, and TPV in 60 random subjects (30 subjects per group) were measured twice by a radiologist (S.R with 4-year of experience performing coronary CT angiography) at a 2-week interval. Another investigator (W.J., with 7-year of experience performing coronary CT angiography) reanalyzed the measurement results of the software (cvi42). The twice measurement results of the first investigators were used to assess intra-observer variability. The measurement results of the two investigators were used to assess inter-observer variability.

## Statistical analysis

Clinical and imaging data were stratified based on follow-up LDL-C status. Categorical variables are expressed as frequency (%) and compared using the Chi-square test or fisher’s exact test (if the expected cell value was ≤ 5). Continuous variables are expressed as the mean ± standard deviation for normally distributed data or the median (interquartile range) for non-normally distributed data. Comparisons between LDL-C controlled and uncontrolled groups were performed using the independent t-test or Mann–Whitney U test as appropriate. Baseline and follow-up CCTA quantitative analysis parameters, such as CACs, TPV, CPV, NCPV, and LD-NCPV, were compared by Wilcoxon signed-rank test. Univariate and multivariate linear regression analyses were performed to determine the impactors of the annual change of TPV, CPV, NCPV, and LD-NCPV. To explore the association between CACs increase and annual PV change according to LDL-C control status, multivariate linear regression (for per-patient level) models and generalized estimating equations (GEE) (for per-plaque level) were constructed. Variables with p-values ≤ 0.1 in univariate regression analysis and recognized cardiovascular risk factors (age, male sex, hypertension, family history of CAD, smoking history, drinking, HbA1c, LDL-C status, and body mass index) were adjusted in the multivariate models. The statistical analyses were performed using IBM SPSS Statistics for Windows, version 25.0 (IBMCorp., Armonk, NY, USA). A two-sided p-value of < 0.05 was considered statistically significant.

## Results

### Clinical characteristics and lipid profiles

The clinical characteristics and lipid profiles of participants in different follow-up LDL-C statuses are presented in Table 1. There were 208 DM patients included in this study (median 68 years old, 64.9% males, median inter-scan interval 3.0 years). There were 75 in the LDL-C controlled group and 133 in the uncontrolled group. The LDL-C controlled group showed older age than LDL-C controlled group (p < 0.001). LDL-C uncontrolled group had higher total cholesterol (baseline: 4.31(3.64,5.20) vs. 3.73(3.16,4.24), p < 0.001; follow-up: 4.28(3.73,4.92) vs. 2.90(2.56,3.32), p < 0.001) and LDL-C (baseline: 2.46(1.87,3.02) vs. 1.84(1.47,2.45), p < 0.001; follow-up: 2.33(1.99,2.83) vs. 1.37(1.09,1.55), p < 0.001) than LDL-C controlled group. LDL-C uncontrolled group had higher triglycerides than LDL-C controlled group at follow-up (1.34(1.00, 2.11) vs. 1.09(0.81, 1.67), p = 0.005). There were no differences between groups in sex, body mass index (BMI), medication use, other high-risk factors, or inter-scan interval.

**Table 1:** Baseline clinical characteristics and lipid profiles

	Total n = 208	LDL-C status		
		LDL-C controlled n = 75	LDL-C uncontrolled n = 133	p-value
Age, years	68(58.5,76)	72(64.0,80.0)	65(56.5,73.5)	< 0.001
Male, n (%)	135(64.9)	50(66.7)	85(63.9)	0.689
BMI, kg/m2	24.5(22.8,26.6)	24.7(22.9,26.8)	24.3(22.8,26.6)	0.625
Systolic BP, mmHg	134(125,144)	135.5(125,144)	133(124,145)	0.825
Diastolic BP, mmHg	76(70,83)	74(67,80)	78(70,85)	0.009
Hypertension, n (%)	156(75)	60(80)	96(72.2)	0.211
Smoking, n (%)	68(32.7)	22(29.3)	46(34.6)	0.438
CAD family history, n (%)	30(14.4)	12(16)	18(13.5)	0.627
ASCVD Risk Categories				0.378
High risk, n (%)	16(7.7)	4(5.3)	12(9.0)	
Very high risk, n (%)	158(76.0)	61(81.3)	97(72.9)	
Extreme risk, n (%)	34(16.3)	10(13.3)	24(18.0)	
Lipid profile at baseline (mmol/L)				
Total cholesterol	4.14(3.43,4.92)	3.73(3.16,4.24)	4.31(3.64,5.20)	< 0.001
Triglyceride	1.44(1.03,2.08)	1.27(1.0,1.77)	1.48(1.05,2.41)	0.153
HDL‑C	1.18(0.95,1.45)	1.18(0.92,1.44)	1.19(0.95,1.46)	0.512
LDL‑C	2.20(1.64,2.84)	1.84(1.47,2.45)	2.46(1.87,3.02)	< 0.001
Glucose, mmol/L	6.91(5.62,8.42)	6.72(5.60,8.24)	7.03(5.71,8.46)	0.583
HbA1C, %	6.9(6.2,7.8)	6.90(6.30,7.60)	6.90(6.20,8.00)	0.908
Cre, μmol/L	74.7(64.1,88.1)	75.00(65.55,88.05)	73.30(63.75,88.50)	0.842
Lipid profile at follow-up (mmol/L)				
Total cholesterol	3.76(3.21,4.56)	2.90(2.56,3.32)	4.28(3.73,4.92)	< 0.001
Triglyceride	1.24(0.94,1.95)	1.09(0.81,1.67)	1.34(1.00,2.11)	0.005
HDL‑C	1.18(0.94,1.42)	1.07(0.88,1.38)	1.24(0.96,1.44)	0.107
LDL‑C	1.99(1.55,2.63)	1.37(1.09,1.55)	2.33(1.99,2.83)	< 0.001
Glucose, mmol/L	6.67(5.59,8.43)	6.73(5.48,8.46)	6.61(5.71,8.43)	0.960
HbA1C, %	6.8(6.2,7.8)	7.00(6.20,7.63)	6.70(6.25,7.80)	0.945
Cre, μmol/L	75.00(63.00,88.00)	79.00(65.00,87.45)	72.5(60.75,88.00)	0.203
Anti-diabetic treatment				
Insulin, n (%)	42(20.2)	19(25.4)	23(17.3)	0.165
Metformin, n (%)	39(18.8)	15(20)	24(18.1)	0.729
Sulfonylurea, n (%)	47(22.6)	18(24)	29(21.8)	0.606
α-glucosidase inhibitor, n (%)	66(31.7)	29(38.7)	37(27.8)	0.137
Other, n (%)	5(2.4)	2(2.7)	3(2.3)	0.594
Non-drug, n (%)	55(26.4)	15(20)	40(30.1)	0.114
Aspirin/clopidogrel, n (%)	110(52.8)	46(61.3)	64(48.1)	0.067
Inter-scan period, year	3.0(1.8,4.3)	3.3(1.6,4.5)	2.9(1.8,4.3)	0.227

Data are reported as n (%) or median (interquartile range) appropriately

BMI: body mass index; CAD: coronary artery disease; ASCVD: atherosclerotic cardiovascular disease; LDL-C: low-density lipoprotein cholesterol; HDL-C: high-density lipoprotein-cholesterol; Cre: creatinine; HbA1C: hemoglobin AIc

### Comparison of CCTA quantitative analysis results between LDL-C controlled and uncontrolled groups on a per-patient level

A detailed overview of CCTA quantitative analysis results is provided in Tables 2, 3. At the per-patient level, CACs, TPV, CPV, NCPV, LD-NCPV, and obstructive patients were increased during follow-up period in the two groups (all p < 0.05). Comparison with LDL-C uncontrolled group, larger CPV were found in LDL-C controlled group at baseline (12.66(2.19, 43.67) vs. 7.61(0, 36.99), p = 0.047), and this difference was not found at follow-up (13.87(1.86,67.88) vs. 30.6(6.16,71.42), p = 0.154). No difference was found on obstructive CAD between groups at baseline and follow-up (p = 0.501 for baseline, p = 0.624 for follow-up). No differences were found in the annual change of CACs, TPV, CPV, NCPV, and LD-NCPV between the two groups (p = 0.253, p = 0.093, p = 0.647, p = 0.157, and p = 0.681 respectively).

**Table 2:** CCTA findings at baseline and follow-up according to LDL-C status

	LDL-C controlled		p	LDL-C uncontrolled		p	p-value	
	baseline	FU		baseline	FU		baseline	FU
On per-patient level								
CACs	61.94(7.96,151.94)	163.79(43.36,345.75)	< 0.001	29.02(0,130.38)	74.58(10.43,273.41)	< 0.001	0.123	0.122
Quantitative CCTA analysis								
TPV, mm^3^	64.61(8.51,227.12)	148.00(53.2,262.55)	< 0.001	78.89(204.94,6.65)	123.42(27.05,287.98)	< 0.001	0.756	0.297
CPV, mm^3^	12.66(2.19,43.67)	30.6(6.16,71.42)	< 0.001	7.61(0,36.99)	13.87(1.86,67.88)	< 0.001	0.047	0.154
NCPV, mm^3^	35.64(0,161.89)	82.61(0,194.76)	0.028	12.90(0.00,159.45)	101.38(0,186.03)	< 0.001	0.643	0.626
LD-NCPV, mm^3^	0(0,23.76)	11.46(0,30.77)	< 0.001	3.29(0,26.06)	11.2(0,35.27)	0.002	0.505	0.801
Obstructive CAD	28(37.3)	41(54.7)	0.002	56(42.1)	68(51.1)	0.004	0.501	0.624
On per-plaque level								
CACs	27.49(6.39,65.75)	37.92(14.00,99.39)	< 0.001	22(4.39,72.63)	32.51(8.74,91.72)	< 0.001	0.549	0.194
Quantitative CCTA analysis								
TPV, mm^3^	25.53(5.85,87.9)	34.68(7.03,92.92)	< 0.001	34.57(6.12,115.78)	24.58(3.81,111.86)	< 0.001	0.46	0.507
CPV, mm^3^	6.32(1.57,18.2)	7.3(1.61,22.2)	< 0.001	5.81(1.10,18.24)	5.81(1.66,18.9)	< 0.001	0.16	0.413
NCPV, mm^3^	0(0,99.03)	0(0,78.89)	0.021	0(0,73.68)	0(0,82.8)	< 0.001	0.204	0.814
LD-NCPV, mm^3^	0(0,4.33)	0(0,9.25)	< 0.001	0(0,6.75)	0(0,11.24)	0.001	0.149	0.952
Type of plaque							0.604	0.123
Calcific	93(57.8)	116(52.7)		141(52.2)	208(56.9)			
Non-calcific	13(8.1)	15(6.8)		31(11.5)	36(9.9)			
Mix	55(34.2)	89(40.5)		98(36.3)	121(33.2)			
Stenosis	20.32(0,45.73)	33.37(20.81,55.07)	< 0.001	25.80(0,44.70)	38.25(22.19,55.81)	< 0.001	0.594	0.440
New plaque		59(26.7)			95(26.0)			0.860
Obstructive lesion	43(26.7)	71(32.3)	< 0.001	80(29.6)	119(32.6)	< 0.001	0.531	0.981

Data are reported as n (%) or median (interquartile range) appropriately

LDL-C: low-density lipoprotein cholesterol; FU: follow up; CACs: coronary artery calcification scores; TPV: total plaque volume; CPV: calcified plaque volume; NCPV: non-calcified plaque volume; LD-NCPV: low-density non-calcified plaque volume; CAD: coronary artery disease

**Table 3:** Comparison of annual changes of CAC and compositional PV between LDL-C controlled and uncontrolled groups.

	LDL-C controlled	LDL-C uncontrolled	p-value
On per-patient level			
CACs change, /year	17.96(4.06,42.97)	10.43(1.32,42.30)	0.253
TPV, mm^3^/year	9.7(0,32.51)	2.28(-1.71,27.88)	0.093
CPV, mm^3^/year	2.65(0,10.10)	1.27(0,6.72)	0.647
NCPV, mm^3^/year	0(− 0.99,21.97)	4.25(0,20.74)	0.157
LD-NCPV, mm^3^/year	0.088(0,3.85)	0(0,4.37)	0.681
On per-plaque level			
CACs change, /year	5.33(0.84,12.28)	4.4(0.32,12.57)	0.474
TPV, mm^3^/year	1.63(0.06,9.75)	0.80(0,8.07)	0.615
CPV, mm^3^/year	0.91(0,2.92)	0.64(0,2.51)	0.018
NCPV, mm^3^/year	0(0,0.002)	0(0,6.58)	0.036
LD-NCPV, mm^3^/year	0(0,0.58)	0(0,0.66)	0.304

Data are reported as median (interquartile range)

LDL-C: low-density lipoprotein cholesterol; CACs: coronary artery calcification scores; TPV: total plaque volume; CPV: calcified plaque volume; NCPV: non-calcified plaque volume; LD-NCPV: low-density non-calcified plaque volume

### Comparison of CCTA quantitative analysis results between LDL-C controlled and uncontrolled groups on a per-plaque level

At the per-plaque level, there were 431 (LDL-C controlled vs. LDL-C uncontrolled: 161 vs. 270) and 585 (LDL-C controlled vs. LDL-C uncontrolled: 220 vs. 365) plaques at baseline and follow-up. The plaque phenotype was not different between groups at baseline and follow-up (p = 0.604 for baseline, p = 0.123 for follow-up). The incidence of new plaques was not different between groups (26.7% vs. 26.0%, p = 0.860). Obstructive lesions were not different between the group at baseline and follow-up (p = 0.531 for baseline, p = 0.981 for follow-up). CACs, TPV, CPV, NCPV, and LD-NCPV were increased in both groups at follow-up CCTA than baseline (all p < 0.05). No differences were found in CACs and compositional PVs between the two groups at baseline or follow-up (p > 0.05). LDL-C uncontrolled group had less annual change of CPV (0.64(0, 2.51) vs. 0.91(0, 2.92), p = 0.018) and more annual change of NCPV (0(0,6.58), 0(0,0.002), p = 0.036) than LDL-C controlled group.

### Impactors of the annual change of TPV and compositional PVs in statins-treated DM patients

Univariate and multivariate analyses of the annual TPV and compositional PVs change impactors are displayed in supplementary materials (table S3). On the per-patient level, CACs increase was positively associated with annual change of TPV (β = 0.43, p < 0.001), CPV (β = 0.23, p < 0.001), NCPV (β = 0.20, p < 0.001), and LD-NCPV (β = 0.08, p < 0.001). Baseline TPV was negatively associated with annual change of TPV (β = − 0.11, p < 0.001), NCPV (β = − 0.12, p < 0.001), and LD-NCPV (β = − 0.02, p < 0.001), but no association was found with annual change of CPV (β = − 0.006, p = 0.28). Hypertension was negatively associated with the annual change of CPV (β = − 6.67, p = 0.032).

On the per-plaque level, CACs increase was positively associated with annual change of TPV (β = 0.61, p < 0.001), CPV (β = 0.19, p = 0.004), NCPV (β = 0.42, p = 0.006), and LD-NCPV (β = 0.13, p = 0.001). Baseline TPV was negatively associated with annual change of TPV (β = − 0.18, p = 0.008), NCPV (β = − 0.12, p = 0.002), and LD-NCPV (β = − 0.04, p = 0.037), but no association was found with annual change of CPV (β = 0.022, p = 0.053). In addition, baseline LDL-C was positively associated with LD-NCPV (β = − 1.31, p = 0.03). Follow-up LDL-C status was not associated with the annual change of TPV and compositional PVs neither on per-patient nor per-plaque level.

### Effect of follow-up LDL-C status on the association between CACs increase and compositional PVs annual changes

The multivariate analysis results are shown in Table 4. On the per-patient level, the multivariate linear regression model showed that the increase in CACs was associated with annual change of TPV (β = 0.65 and 0.378, respectively, both p < 0.001), CPV (β = 0.169 and 0.232, respectively, p = 0.007 and p < 0.001), NCPV (β = 0.469 and 0.144, respectively, both p = 0.001), and LD-NCPV (β = 0.082 and 0.086, respectively, p = 0.002 and p = 0.004) in LDL-C controlled and LDL-C uncontrolled group.

**Table 4:** Multivariate analysis of the association between annual changes in CACs and annual PVs changes according to LDL-C status

	LDL-C controlled	p	LDL-C uncontrolled	p
	β (95% CI)		β (95% CI)	
Association with annual change in Agatston CACs				
On per-patient level				
TPV	0.650(0.347,0.952)	< 0.001	0.378(0.292,0.464)	< 0.001
CPV	0.169(0.048,0.290)	0.007	0.232(0.206,0.258)	< 0.001
NCPV	0.469(0.207,0.731)	0.001	0.144(0.063,0.226)	0.001
LD-NCPV	0.082(0.032,0.133)	0.002	0.086(0.066,0.106)	0.004
On per-plaque level				
TPV	0.389(0.081,0.715)	0.014	0.413(0.286,0.539)	< 0.001
CPV	0.118(0.074,0.162)	< 0.001	0.226(0.165,0.286)	< 0.001
NCPV	0.268(− 0.037,0.573)	0.085	0.188(0.099,0.276)	< 0.001
LD-NCPV	0.056(− 0.007,0.119)	0.080	0.106(0.066,0.146)	< 0.001

The multivariate analysis models in different samples were adjusted for age, sex, BMI, hypertension, smoking history, drinking history, CAD family history, insulin, baseline CACs and total PV

Abbreviations are as in Table 3

On the per-plaque level, the increase in CACs was associated with the annual change of TPV (β = 0.389 and 0.413, respectively, p = 0.014 and p < 0.001) and CPV (β = 0.118 and 0.226, both p < 0.001) both in LDL-C controlled and LDL-C uncontrolled groups. The increase in CACs was associated with NCPV and LD-NCPV in LDL-C uncontrolled patients (NCPV: β = 0.188, p < 0.001; LD-NCPV: β = 0.106, p < 0.001) but not in LDL-C controlled group (NCPV: β = 0.268, p = 0.085; LD-NCPV: β = 0.056, p = 0.08).

### Intra- and inter-observer reproducibility

Intra- and inter-observer variabilities of CACs and compositional PVs (total, calcific, non-calcific) are shown in supplementary materials (Additional file 1: Table S4). The intra- and inter-observer intraclass correlation coefficients (ICCs) for compositional PVs (total, calcific, non-calcific) between 0.926–1.00 and 0.918–0.999, respectively, and for stenosis, the intra- and inter-observer ICCs were 0.974 and 0.925, respectively.

## Discussion

Our study explored the impact of follow-up LDL-C status on the relationship between CACs increase and componential PVs progression in statins-treated diabetes patients. After adjusting for confounding factors, CACs increasing but not LDL-C status was positively associated with the annual change of TPV, CPV, NCPV, and LD-NCPV both on per-plaque and per-patient levels. The increase CACs was associated with TPV, CPV, NCPV, and LD-NCPV progression regardless of LDL-C status on the per-patient level. The increase of CACs was positively associated with TPV and CPV progression in both LDL-C controlled and uncontrolled groups on the per-plaque level, whereas the CACs increase was positively associated with NCPV and LD-NCPV progression only in LDL-C uncontrolled group.

### Characteristic of CAD in diabetes

It is well known that DM imparts a 2- to threefold increase in the risk of developing coronary artery disease [13]. Hyperglycemia, dyslipidemia, and advanced glycation end-products accumulation associated with DM synergistically contribute to coronary atherosclerosis development [13]. The severity and extent of coronary atherosclerosis in diabetes patients are more severe than in non-diabetic patients [14, 15]. The results of the intravascular imaging study indicated that both the mean percent of calcified area and area composed of necrotic core were more pronounced in diabetic subjects [16]. Part of explanation is that the coronary tree in diabetes is chronically in an inflammatory environment and has a greater number of healed plaque ruptures than in non-DM counterparts.

In recent years, there have been numerous published data on the progression of coronary atherosclerosis in patients with metabolic abnormalities [17–19]. DM has been shown to be associated with rapid progression of coronary plaque, and higher glycosylated hemoglobin was associated with CACs progression [20]. Available evidence suggests that serum LDL-C, an important intermediate metabolite in coronary atherosclerosis formation, is a predictor of CAD in populations with DM, which even exceeding the predictive power of glycosylated hemoglobin [4]. Previous studies reported that intensive LDL-C control in reference to the recommended target goals of LDL-C < 70 mg/dL is associated with a reduced atherosclerosis progression and accelerated calcification progression [21, 22]. This implies that intensive LDL-C control may suggest plaque stabilization and reduced risk. There is no consensus as to whether this is still the case in the diabetic population.

Our study included statins-treated diabetes as subjects, and our data showed that annual progression of CACs and annual progression of TPV were numerically greater in these individuals compared to previous studies. In statins-treated diabetic patients, we found that LDL-C was positively associated with increased plaque volume of low-density non-calcified plaques at the per-plaque level, implying an increased instability of this individual plaque. Even so, our data did not show an independent correlation between follow-up LDL-C status and componential PVs or TPV progression. This may be due to metabolic disturbances and the interaction of multiple concomitant conditions in statins-treated diabetic patients, and the exact mechanism needs to be confirmed by further in-depth studies.

### Coronary artery calcium progression and CAD

Patients with diabetes are more likely to develop diffuse coronary calcification in the coronary tree [23]. Coronary artery calcium (CAC) accompanies the development of advanced atherosclerosis [24]. Society of cardiovascular computed tomography (SCCT) guidelines recommend considering CAC scans among asymptomatic individuals with a risk of ASCVD. Computed tomography derived CAC is a subclinical marker of atherosclerotic plaque burden. The generally accepted view is that the presence and extent of calcium deposits along the coronary arteries help estimate the severity of ASCVD and improve the potential for reclassification of cardiovascular disease risk [25].

Relevant pathological studies have shown that, unlike peripheral vascular disease where calcification primarily affects the medial layer, coronary atherosclerosis is dominantly by intimal calcification [17]. Coronary artery calcification pathologically begins as microcalcifications and grows into fragmented or nodular calcium in the advanced stage [25, 26]. In general, spotty calcification is one of the features of high-risk plaques and is considered to be a predictor of plaque stability. Complete calcification is seen as characteristic of plaque stabilization. It is not clear whether the association of CAC with adverse outcomes is related to the calcified plaque itself as the source of the events or just the calcified plaque predicts the presence of CAD accurately.

### Coronary atherosclerosis in response to statin therapy

Considering DM individuals exhibit multiple concomitant metabolic abnormalities, patients without contraindications are recommended strict lipid-lowering therapy [5]. Statins are a class of HMG-CoA reductase inhibitor that mainly dose-dependently decrease plasma cholesterol. Available evidence suggested the statins have an ameliorative effect on cardiovascular morbidity and mortality. The US preventive services task force recommendation statement (USPSTF) recommended statins use for the primary prevention of cardiovascular disease in adults [27]. Different statins doses and LDL-C control targets were formulated according to different cardiovascular risk stratification [28]. The clinical benefit of statins treatment is mainly driven by the absolute LDL-C reduction. In clinical practice, poor responses to statins treatment in individuals occasionally occur. In addition to the variations in genetic background, it is mainly caused by poor compliance.

It has been established that statins stabilize plaque by promoting calcification in coronary atherosclerotic lesions [29, 30]. Non-invasive images explored the impact of statins on the progression of plaque atherosclerosis in whole-heart evaluations, revealing that statins slow the progression of TPV and promote the progression of CPV meanwhile inducing phenotypic plaque transformation [22, 31, 32]. Further atherosclerosis progression is present in lipid-lowering therapy DM patients, despite the same reached levels of LDL-C as in non-DM patients [33]. Our findings also showed that atherosclerotic plaque progressed at follow-up even in LDL-C controlled diabetic patients. Lee et al. investigated whether the relationships between the increase of CACs and compositional PVs progression differed in statins-treated and non-statins-treated patients [34]. The results showed that CACs progression was negatively correlated with the annual change of noncalcified PV. In contrast to the above studies, our study explored the relationship between increased CACs and plaque progression in statins-treated diabetic patients using LDL-C as an indicator of response to statins therapy. We found that CACs increases were positively related to TPV, CPV, NCPV, and LD-NC PV progression regardless of the LDL-C status on per-patient level. This inconsistent result suggests that the progression of atherosclerotic lesions in statins-treated diabetic patients differs from that in the general population. Complex atherogenic mechanisms in the diabetic population might mitigate the effect of statins on plaque stabilization.

The relationship of CACs to plaque instability is exceptionally complex. Calcification correlates with entire atherosclerosis plaque burden and, in some cases, with stable plaques. The aggregation of changes in individual lesions for patient assessment could not comprehensively evaluate the impact of statins on individual coronary atherosclerotic lesions. Our study conducted a comprehensive analysis at the per-plaque level to explore the relationship between increased CACs and PV progression in statins-treated diabetic patients. The data showed that increased CACs were positively associated with the progression of NCPV and LD-NCPV at the per-plaque level in LDL-C uncontrolled patients but not in the LDL-C controlled patients. This result appears to be inconsistent with the results at the per-patient level. After neglecting interactions between coexisting plaques, the CACs increase is accompanied by atherosclerosis lipid or necrotic cores development in statins-treated DM patients with LDL-C uncontrolled at follow-up. It might be one of the potential mechanisms of formation of culprit lesions leading to long-term acute coronary events in these populations.

The present study still had some limitations. First, this is an observational single-center study, and the preliminary results need to be validated by a larger-sample of multi-center study. Secondly, the baseline LDL-C might affect the result. We adjusted for the baseline LDL-C in the multivariate analysis to ensure the reliability of our result. Thirdly, the current study did not include follow-up data. Whether the annual progression of non-calcific PV in individual lesions induces long-term adverse cardiovascular events is unclear. This issue deserves further in-depth study.

## Conclusions

In statins-treated diabetic patients, increased CACs were positively associated with compositional PVs progression regardless of LDL-C status. CACs increases were not associated with progression of NCPV and LD-NCPV in individual plaques in LDL-C controlled diabetic patients, but it was accompanied by individual plaques NCPV and LD-NCPV progression in the LDL-C uncontrolled counterpart. The findings suggest that increased CACs indicate the progression of compositional PVs and might be accompanied by increased instability of individual plaques in statins-treated DM patients with uncontrolled LDL-C.

## Supplementary Information

** Additional file 1: Table S1. **ASCVD Risk Categories and LDL-C Treatment Goals. **Table S2.** CT scanning parameters. **Table S3.** Association between risk factors, CAC increase and annual increase in total, calcified, noncalcified, and low-density noncalcified plaque volume. **Table S4.** Inter- and intra-observer variability of CACs and compositional PV.

## Data Availability

The datasets generated and/or analyzed during the current study are available from the corresponding author upon reasonable request.

## Abbreviations

CACs: Coronary artery calcium score
CAD: Coronary artery disease
ASCVD: Atherosclerotic cardiovascular disease
LDL-C: Low-density lipoprotein cholesterol
TPV: Total plaque volume
CPV: Calcified plaque volume
NCPV: Non-calcified plaque volume
LD-NCPV: Low-density non-calcified plaque volume
