# Supplementary material for: The effect of LDL-C status on the association between increased coronary artery calcium score and compositional plaque volume progression in statins-treated diabetic patients: evaluated using serial coronary CTAs
Source: Cardiovasc Diabetol. 2022 Jun 30;21:121. doi: 10.1186/s12933-022-01556-y (PMC9248151; doi:10.1186/s12933-022-01556-y)
Supplement: Supplementary file 1 — Additional file 1: Table S1. ASCVD Risk Categories and LDL-C Treatment Goals. Table S2. CT scanning parameters. Table S3. Association between risk factors, CAC increase and annual increase in total, calcified, noncalcified, and low-density noncalcified plaque volume. Table S4. Inter- and intra-observer variability of CACs and compositional PV. [file 12933_2022_1556_MOESM1_ESM.docx]

**Table S1 ASCVD Risk Categories and LDL-C Treatment Goals**

| **Risk category** | **LDL-C treatment goals(mg/dL)** |
| --- | --- |
| Extreme risk | <55 |
| Very high risk | <70 |
| High risk | <100 |
| Moderate risk | <100 |
| Low risk | <130 |

Abbreviations: Atherosclerotic cardiovascular disease; LDL-C: low-density lipoprotein cholesterol

**Table S2 CT scanning parameters**

| **Parameters** | **SOMATOM Definition** | **SOMATOM Definition FLASH** | **Revolution CT** |
| --- | --- | --- | --- |
| **Machine type** | Siemens Medical Solutions, Forchheim, Germany | Siemens Medical Solutions, Forchheim, Germany | GE Healthcare, Waukesha, WI USA |
| **tube voltage** | 120kV | 120 kV | 100-120kV (kV Assist) |
| **tube current** | 220mAs | 220mAs | 500-600 mA (Smart mA) |
| **Contrast medium** | Non-ionic iodinated | Non-ionic iodinated | Non-ionic iodinated |
| dose | 0.9mL/Kg | 0.9mL/Kg | 0.9mL/Kg |
| flow rate | 5ml/s | 5ml/s | 5ml/s |
| **collimation** | 64/128 0.5mm | 64/128 0.5mm | 256 0.625mm |
| **rotation time** | 0.33-0.4s | 0.33-0.4s | 0.28s |
| **Gating protocol** | Retrospective/prospective | Retrospective/prospective | Retrospective/prospective |
|  |  |  |  |

**Table S3 Association between risk factors, CAC increase and annual increase in total, calcified, noncalcified, and low-density noncalcified plaque volume**

|  | **TPV** | | | | **CPV** | | | | **NCP** | | | | **LD-NCP** | | | |
| --- | --- | --- | --- | --- | --- | --- | --- | --- | --- | --- | --- | --- | --- | --- | --- | --- |
|  | **univariable** | | **multivariable** | | **univariable** | | **multivariable** | | **univariable** | | **multivariable** | | **univariable** | | **multivariable** | |
|  | **β** | **p** | **β** | **p** | **β** | **p** | **β** | **p** | **β** | **p** | **β** | **p** | **β** | **p** | **β** | **P** |
| **Per-patient** |  |  |  |  |  |  |  |  |  |  |  |  |  |  |  |  |
| Age | 0.37 | 0.37 | 0.33 | 0.41 | -0.04 | 0.806 | -0.039 | 0.75 | 0.41 | 0.23 | 0.38 | 0.27 | 0.08 | 0.33 | 0.07 | 0.39 |
| Sex | 6.81 | 0.47 | 11.28 | 0.25 | 6.23 | 0.124 | -1.99 | 0.51 | 1.14 | 0.88 | 13.44 | 0.11 | 1.03 | 0.57 | 2.37 | 0.21 |
| BMI | -0.44 | 0.74 | -1.09 | 0.37 | 0.64 | 0.259 | 0.17 | 0.65 | -1.09 | 0.31 | -1.28 | 0.22 | -0.16 | 0.53 | -0.14 | 0.54 |
| Hypertension | -4.21 | 0.69 | 0.86 | 0.93 | -4.63 | 0.034* | -6.67 | 0.032* | -0.41 | 0.96 | 6.84 | 0.43 | -1.54 | 0.45 | 1.17 | 0.55 |
| Smoking | 7.41 | 0.44 | -0.90 | 0.93 | 9.36 | 0.023 | 3.95 | 0.21 | -0.87 | 0.91 | -3.66 | 0.68 | 0.38 | 0.84 | -2.79 | 0.16 |
| drinking | 17.81 | 0.16 | -2.85 | 0.83 | 14.69 | 0.006 | 1.07 | 0.79 | 3.87 | 0.71 | -3.06 | 0.79 | 2.80 | 0.25 | 0.77 | 0.77 |
| CAD family history | 1.52 | 0.91 | -15.61 | 0.21 | 7.93 | 0.150 | -6.47 | 0.09 | -5.89 | 0.58 | -8.97 | 0.40 | 0.16 | 0.95 | -0.92 | 0.70 |
| CACs, /year | 0.35 | <0.001* | 0.43 | <0.001* | 0.23 | <0.001* | 0.23 | <0.001* | 0.12 | 0.003* | 0.20 | <0.001* | 0.07 | <0.001* | 0.08 | <0.001* |
| HbA1c2 | -0.11 | 0.98 | -1.13 | 0.69 | 0.13 | 0.931 | -1.27 | 0.14 | -0.32 | 0.91 | 0.009 | 0.997 | -0.26 | 0.70 | -0.39 | 0.47 |
| LDL-C status | -16.17 | 0.09* | -7.07 | 0.44 | -2.12 | 0.60 | -0.65 | 0.82 | -9.32 | 0.06* | -6.71 | 0.39 | -1.20 | 0.51 | 0.25 | 0.89 |
| Baseline TPV | -0.04 | 0.04* | -0.11 | <0.001* | 0.03 | <0.001* | 0.006 | 0.28 | -0.08 | <0.001* | -0.12 | <0.001* | -0.10 | 0.008* | -0.02 | <0.001* |
| Baseline LDL-C | -4.16 | 0.47 | - | - | -4.73 | 0.052 | - | - | 0.71 | 0.88 | - | - | 0.42 | 0.69 | - | - |
| **Per-plaque** |  |  |  |  |  |  |  |  |  |  |  |  |  |  |  |  |
| Age | 0.44 | 0.38 | 0.15 | 0.58 | 0.03 | 0.537 | 0.016 | 0.64 | 0.39 | 0.41 | 0.12 | 0.65 | 0.10 | 0.42 | 0.04 | 0.57 |
| Sex | 1.59 | 0.75 | -4.69 | 0.31 | -2.25 | 0.138 | -1.32 | 0.36 | 3.64 | 0.40 | -3.61 | 0.37 | 1.46 | 0.195 | -0.06 | 0.96 |
| BMI | -0.68 | 0.30 | -0.59 | 0.36 | -0.09 | 0.644 | -0.20 | 0.30 | -0.58 | 0.30 | -0.41 | 0.47 | -0.08 | 0.53 | -0.07 | 0.65 |
| Hypertension | -4.66 | 0.71 | 0.53 | 0.95 | 4.38 | 0.254 | 3.19 | 0.32 | -9.21 | 0.40 | -3.03 | 0.58 | -1.15 | 0.68 | -0.03 | 0.98 |
| Smoking | 5.24 | 0.50 | 1.73 | 0.77 | 0.66 | 0.677 | 0.87 | 0.57 | 4.77 | 0.52 | 1.32 | 0.81 | 1.79 | 0.34 | 0.62 | 0.63 |
| drinking | 1.70 | 0.88 | 2.08 | 0.78 | 0.26 | 0.900 | 0.98 | 0.55 | 1.68 | 0.85 | 1.28 | 0.85 | 0.46 | 0.84 | -0.38 | 0.85 |
| CAD family history | 16.70 | 0.27 | 11.87 | 0.117 | 1.99 | 0.208 | 3.41 | 0.078 | 13.81 | 0.35 | 7.46 | 0.29 | 3.49 | 0.35 | 2.08 | 0.26 |
| CACs, /year | 0.40 | 0.02* | 0.61 | <0.001* | 0.20 | 0.007* | 0.19 | 0.004* | 0.20 | 0.28 | 0.42 | 0.006* | 0.079 | 0.079 | 0.13 | 0.001* |
| HbA1c2 | -2.92 | 0.36 | -3.59 | 0.095 | 0.35 | 0.477 | -0.55 | 0.43 | -3.28 | 0.29 | -3.07 | 0.09 | -0.85 | 0.19 | -0.94 | 0.008* |
| Baseline TPV | -0.16 | 0.06* | -0.18 | 0.008* | 0.03 | 0.04* | 0.022 | 0.053* | -0.19 | 0.012 | -0.20 | 0.002* | -0.04 | 0.10 | -0.04 | 0.037* |
| LDL-C status | 2.77 | 0.64 | 2.16 | 0.67 | 1.21 | 0.62 | 1.139 | 0.57 | 1.92 | 0.71 | 1.36 | 0.74 | 0.33 | 0.79 | 0.84 | 0.42 |
| baseline LDL-C | 2.89 | 0.28 | - | - | -0.69 | 0.15 | - | - | 3.37 | 0.20 | - | - | 1.01 | 0.08* | 1.31 | 0.030* |

Abbreviations: LD-NCP: low-density non-calcific plaque; CP: calcific plaque; NCP: non-calcific plaque; BMI: body mass index; CAD: coronary artery disease; LDL-C: low-density lipoprotein cholesterol; HbA1C: hemoglobin AIc.

**Table S4 Inter- and intra-observer variability of CACs and compositional PV**

|  | intra-observer ICC (95%CI) | p value | inter-observer ICC (95%CI) | p value |
| --- | --- | --- | --- | --- |
| TPV | 0.992(0.981,0.997) | <0.001 | 0.989(0.974,0.995) | <0.001 |
| CP | 1(1,1) | <0.001 | 0.999 (0.998,1) | <0.001 |
| NCP | 0.989(0.974,0.995) | <0.001 | 0.986 (0.968,0.994) | <0.001 |
| LD-NCP | 0.926(0.829,0.969) | <0.001 | 0.918(0.815,0.965) | <0.001 |
| Stenosis | 0.974(0.942,0.989) | <0.001 | 0.925(0.837,0.967) | <0.001 |

Abbreviations: ICC, intraclass correlation coefficient; CI: confidence interval; LD-NCP: low-density non-calcific plaque; CP: calcific plaque; NCP: non-calcific plaque.
